# Research Progress of Water Treatment Technology Based on Nanofiber Membranes

**DOI:** 10.3390/polym15030741

**Published:** 2023-01-31

**Authors:** Keyu Ji, Chengkun Liu, Haijun He, Xue Mao, Liang Wei, Hao Wang, Mengdi Zhang, Yutong Shen, Runjun Sun, Fenglei Zhou

**Affiliations:** 1 School of Textile Science and Engineering, Xi’an Polytechnic University, Xi’an 710048, China; 2 Key Laboratory of Functional Textile Material and Product of the Ministry of Education, Xi’an Polytechnic University, Xi’an 710048, China; 3 Shaanxi College Engineering Research Center of Functional Micro/Nano Textile Materials, Xi’an Polytechnic University, Xi’an 710048, China; 4 Engineering Research Center for Knitting Technology of the Ministry of Education, Jiangnan University, Wuxi 214122, China; 5 Centre for Medical Image Computing, Department of Medical Physics and Biomedical Engineering, University College London, London WC1E 6BT, UK

**Keywords:** water treatment, membrane separation technology, electrospinning, nanofiber membrane, antifouling technology

## Abstract

In the field of water purification, membrane separation technology plays a significant role. Electrospinning has emerged as a primary method to produce nanofiber membranes due to its straightforward, low cost, functional diversity, and process controllability. It is possible to flexibly control the structural characteristics of electrospun nanofiber membranes as well as carry out various membrane material combinations to make full use of their various properties, including high porosity, high selectivity, and microporous permeability to obtain high-performance water treatment membranes. These water separation membranes can satisfy the fast and efficient purification requirements in different water purification applications due to their high filtration efficiency. The current research on water treatment membranes is still focused on creating high-permeability membranes with outstanding selectivity, remarkable antifouling performance, superior physical and chemical performance, and long-term stability. This paper reviewed the preparation methods and properties of electrospun nanofiber membranes for water treatment in various fields, including microfiltration, ultrafiltration, nanofiltration, reverse osmosis, forward osmosis, and other special applications. Lastly, various antifouling technologies and research progress of water treatment membranes were discussed, and the future development direction of electrospun nanofiber membranes for water treatment was also presented.

## 1. Introduction

**Figure 1:**
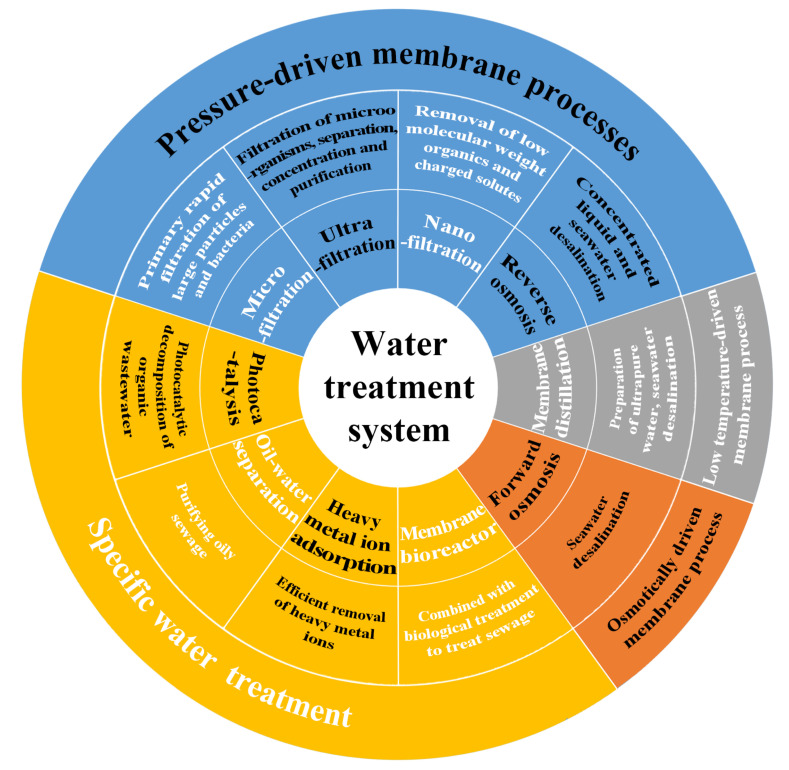
Classification of membrane separation techniques in water treatment systems.

Water pollution is one of the most urgent environmental issues among the different pollution risks, having a direct negative impact on human health and other biological populations [1,2]. The key to properly addressing the issues of water pollution and water scarcity is to treat and recycle wastewater [3]. The research has reported that there are various methods for treating wastewater, including traditional water treatment techniques such as oxidation, electrocoagulation, and chlorination [4,5,6], which are time-consuming, energy consuming, and may lead to secondary pollution. However, membrane technology can effectively solve these problems. Due to its advantages in water treatment, such as a lack of secondary contaminants, high selectivity, high separation efficiency, and superior stability, membrane technology has received a lot of attention [7,8,9,10]. As shown in Figure 1, membrane separation techniques include pressure-driven membrane processes (microfiltration, ultrafiltration, nanofiltration, reverse osmosis), low-temperature-driven membrane processes, and several other processes used in special fields (forward osmosis, membrane bioreactor, heavy metal ion adsorption, oil–water separation, and photocatalysis), and some of these technologies have recently been widely used for water separation and purification [11,12,13,14,15,16]. The selection of materials and preparation methods for these membranes determines the performance and economic advantages of the nanofiber-membrane-based water treatment process.

Nanofiber membranes have a range of properties including a large specific surface area, high porosity, ease of modification, and strong compatibility with other functional materials and have significant potential in solving water pollution problems [17,18,19,20,21]. Among fiber-spinning technologies, electrospinning is a straightforward method for producing continuous and uniform nanofibers from polymers. Researchers have successfully created nanofiber membrane products from hundreds of polymers using this technique [22]. Therefore, electrospinning has attracted wide attention in recent decades and has been widely used to prepare nanofiber membranes with various functions [23,24]. Due to their microporous penetrability, electrospun nanofiber membranes can overcome some of the inherent drawbacks of traditional membranes, such as their poor permeation flux and weak antifouling capabilities [25,26]. As a result, the development of electrospinning technology for the production of nanofiber membranes with high flux, superior antifouling and mechanical qualities, and microporous penetrability for water treatment has been a popular research focus [27].

Nowadays, the main challenge researchers are facing in water treatment membranes is to create high-permeability nanofiber membranes with antifouling performance, strong physical and chemical properties, and long-term stability. The management of the diameter, pore size, porosity, and mechanical strength of electrospun nanofibers is a crucial component of improving the interception and flux of the fiber membrane as well as its service life [28,29]. The membrane filtration system is composed of four elements: the water phase and gas phase on both sides of the membrane, the membrane interface, and the driving force. An efficient, stable, and energy-saving water filtration process can be realized by coordinating these four elements reasonably [30]. This paper systematically reviews 10 different applications of membranes in water treatment, including their principles, challenges, research progress, and development direction, and also discusses the anti-fouling ability of membranes, membrane cleaning, and the means of membrane flux recovery, which are different from other relevant reviews. It is hoped that this review can provide some insights and theoretical references for research in the field of water treatment. Table 1 shows the abbreviations of some words and phrases used in this paper.

## 2. Technology for Routine Water Purification Based on Nanofiber Membranes

With the continuous development and functionalization of nanofiber membranes, a growing number of researchers are committed to exploring the practical application of nanofibers in membrane separation technology. Generally, membrane treatment processes can be divided into pressure-driven, osmotically driven, and low temperature-driven processes. Table 2 summarizes the characteristics of different membrane processes. The main pollutants in wastewater should be removed by different membrane processes under the applicable conditions of different types of membranes.

### 2.1. Pressure-Driven Membrane Processes

**Figure 2:**
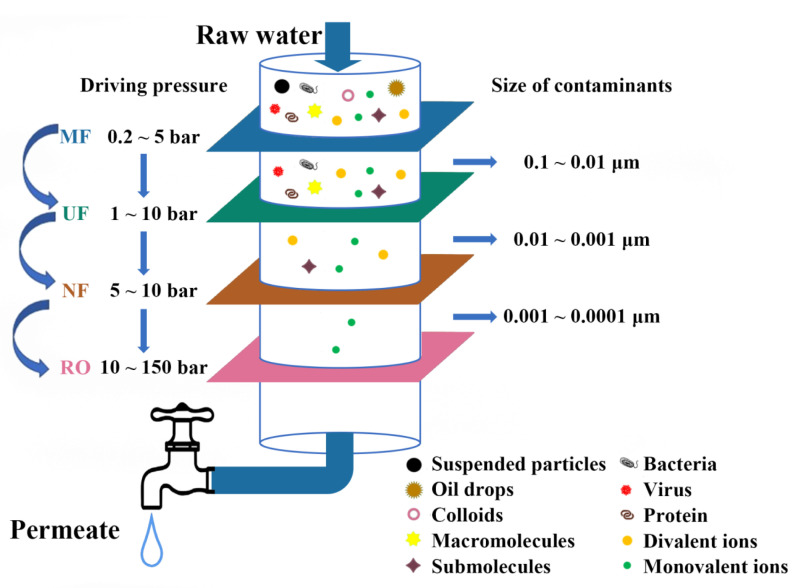
Schematics of a water treatment system based on pressure-driven membranes.

Pressure-driven membrane processes include MF, UF, NF, and RO, which use the pressure applied on the feed side of the filter as the driving force to divide the water into two parts: purified water (permeate) and concentrated liquid (retentate). Each membrane exerts a distinct amount of pressure and has a pore size range, specific permeability, and selectivity for efficient water treatment. The rejection characteristics of different pressure-driven membrane technologies in water treatment are shown in Figure 2 [37,38].

#### 2.1.1. Microfiltration (MF)

MF is the earliest membrane filtration technology, which is a sieve separation process under the effect of differential pressure. The MF membrane has pores that range from 0.1 to 10 μm in size and can effectively filter suspended particles with diameters greater than 0.1 μm [39]. The MF membrane’s filtration procedure is more similar to coarse filtration, which is generally the first part of the water treatment system.

Compared with MF membranes fabricated by other methods, ENMs can optimize the sub-micron pore size to a few micrometers or even lower, which can greatly improve the rejection. In addition, ENM is superior to the conventional MF membrane in many aspects such as its higher porosity, connected pores, and thinner thickness [40,41,42].

**Figure 3:**
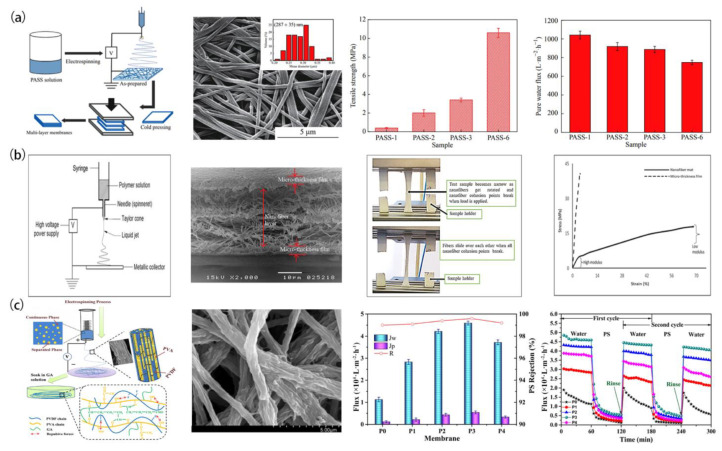
Preparation process, microstructure, and properties of MF membranes: (**a**) PASS membrane. Reprinted with permission from Ref. [47]. Copyright 2019 Springer Nature. (**b**) PVC membrane. Reprinted with permission from Ref. [48]. Copyright 2020 SAGE Publications. (**c**) PVDF/PVA membrane. Reprinted with permission from Ref. [49]. Copyright 2019 John Wiley and Sons, Inc.

Nanofiber membranes subject to high flux and high driving pressure may be separated or broken, resulting in irreversible physical damage. Therefore, the emphasis has been focused on the improvement of the mechanical properties of nanofiber membranes [43,44,45]. The majority of researchers used micro/nanofiber composite membranes, multi-layer superimposition, or surface modification and other methods to improve the mechanical performance [46,47,48]. For example, on the basis of ensuring the filtration performance, the use of nylon fabric as the substrate is a typical research strategy to enhance the mechanical qualities [46]. Additionally, in the case of multilayer ENMs as shown in Figure 3a, there was less than 20% loss of water flux while maintaining strong mechanical characteristics and a high interception rate [47]. Furthermore, the method of surface coating can also solve the mechanical problem to a certain extent. When coated on the electrospun PVC nanofiber felt, the adhesion between fibers will effectively enhance the strength of the membrane (Figure 3b), and the increase in fiber density can also improve the rejection rate of the membrane [48].

On the other hand, water flux is another research focus regarding MF membranes. Increasing the hydrophilicity of MF membranes can improve the water flux of membranes to a certain extent [50,51]. As a typical high-strength fiber material, hydrophilic modification of PVDF and its application in water treatment has always been a broad research topic [49,50,51]. A hybrid nanofiber film with a ridge valley structure and a bicontinuous phase was obtained by electrospinning by blending PVA into PVDF (Figure 3c). Its rough fiber surface endows it good hydrophilicity and super wettability (contact angle of 74.5°), leading to the improvement of the water flux of the membrane [49]. In addition, surface coating is another way to make PVDF hydrophilic. For example, the CNFs modified by Meldrum’s acid were coated on PVDF ENMs to obtain hydrophilic ENMs. The water contact angles dropped from 142.6° to 121.0° after hydrophilic modification, indicating that the wettability of the nanofiber membrane with CNF coating was improved compared with that of PVDF ENM and made the water flux increase significantly [51].

The use of MF membranes often requires a large driving force, which may lead to mechanical damage of membranes. Therefore, mechanical strengthening of nanofibers and physical adhesion between their composite membranes are the key issues to be solved. In addition, when the suspended particles in the water are small, it is easy to cause membrane pore plugging and to reduce the water flux due to the large pore size of the MF membrane. In order to obtain the best filtration performance, the pore size of the membrane should be controlled to be slightly smaller than the size of the particles. Therefore, it is particularly important to control the electrospinning process in production to prepare MF membranes with appropriate pore sizes.

#### 2.1.2. Ultrafiltration (UF)

Water molecules and small solute particles in the feeding solution pass through the membrane during the UF membrane separation process, whereas large particles or macromolecules with a diameter from 10 to 100 nm are retained [33]. UF is the primary method for removing colloids, germs, and viruses from water and has wide application in potable water treatment, reclamation, medical, concentration, and wastewater treatment fields [52,53,54].

The conventional UF membrane is mainly prepared by phase inversion with a wide pore size distribution, which tends to aggravate membrane pollution, thus damaging the stability of the membrane [55,56]. The high porosity and small pore size of ENMs are exactly what UF membranes need. A UF membrane prepared by a nanofiber composite not only had a high water flux (>130 L·m^−2^·h^−1^) but also had a high retention rate (>99.9%), surpassing the conventional UF membrane [56].

In addition, UF is often used for the separation of biological solutions, such as high-purity biomolecular purification [57] and the removal of microorganisms in the fermentation broth [58]. A PEO/NaAlg layer cross-linked with calcium chloride was coated onto electrospun ε-caprolactone nanofibers to prepare a new biomass separation membrane, which can effectively allow the permeation of plasmid DNA and separate suspended solids and RNA. Although both centrifugation and UF could remove microorganism cells, the removal rate of UF (86%) was higher than that of centrifugation (53%) [58]. Some researchers have carried out a study on the recovery of surfactin from fermentation broths in cross-flow UF by using PES UF membranes [59]. The results showed that the recovery rate of surfactin could reach 75% in the UF process. Further, the PSF UF membrane was combined with an electrodeionization (EDI) membrane to purify xylitol prepared by microbial fermentation [60]. This UF–EDI membrane configuration could remove 99% of microorganisms, biomass, pigments, and ionic impurities.

**Figure 4:**
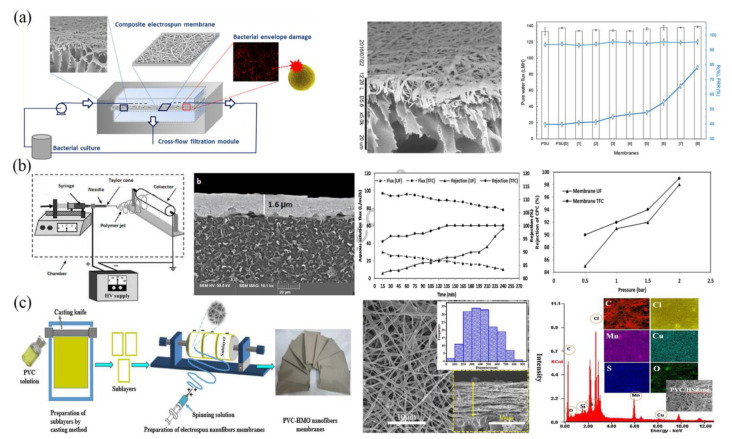
Preparation process, microstructure, and properties of UF membranes: (**a**) PAA/PVA-PSF membrane. Reprinted with permission from Ref. [64]. Copyright 2018 American Chemical Society. (**b**) PEI/PAN membrane. Reprinted with permission from Ref. [65]. Copyright 2017 Elsevier Ltd. (**c**) PVC/MnO membrane. Reprinted with permission from Ref. [66]. Copyright 2020 Elsevier Ltd.

Due to the increasing usage of UF membranes in the food and medical industries in recent years, there are strict criteria for the membranes’ antibacterial qualities. Through electrospinning, a variety of antibacterial substances can be added into the solution to create a UF membrane with antibacterial capabilities [61,62,63]. In order to create a composite UF membrane with a hydrophilic surface and antibacterial ability, researchers prepared a PAA/PVA nanofiber composite membrane through electrospinning and crosslinking and then covered it on the surface of a PSF membrane (Figure 4a). This UF membrane had strong hydrophilicity, which greatly reduced pollution caused by organic materials (the membrane flux recovery rate reached 80.2%). Moreover, its antibacterial effectiveness against *Staphylococcus aureus* was greatly increased compared to the pure PSF membrane due to the fact that PAA has a chelating impact on the bivalent cations of bacterial membranes [64].

The development of more novel nanofiber composite membranes for UF can broaden their application directions. For example, recent studies have focused on the removal of salt ions by UF membranes. Some researchers combined PAN ENM and PET non-woven fabrics and used cetylpyridinium chloride for surface modification to prepare a novel composite membrane, which could successfully adsorb arsenate ions and was 1.1–1.3 times more efficient in rejecting arsenate ions than the conventional UF membrane (Figure 4b) [65]. Additionally, hydrous manganese oxide (HMO) nanoparticles (NPs) were modified by a mercaptosilane coupling agent and then directly embedded into PVC nanofiber by electrospinning to create a nanofiber membrane as shown in Figure 4c. The composite membrane containing modified adsorbents had a strong chelating ability and a high affinity for salt ions with rejection rates for Cu(II) and Ni(II) of 90% and 80%, respectively. Moreover, it still showed a good removal performance of metal salt ions after four consecutive adsorption–desorption cycles [66].

To sum up, a desirable UF nanofiber membrane should be a porous material with excellent mechanical properties and chemical stability. Many studies have shown that hydrophilic UF membranes often have better antifouling effects than hydrophobic ones. Hydrophilic modification methods and optimization of the membrane structure and membrane top coating should be further studied to enhance the hydrophilicity, permeability, and antifouling properties of UF membranes.

#### 2.1.3. Nanofiltration (NF)

NF is an energy-saving, environmentally friendly, and simple separation technology with the unique ability to remove low-molecular-weight organics and charged solutes [67,68,69,70]. NF membranes are usually neutral or negatively charged membrane materials [71] with a microporous structure with a pore size less than 2 nm and molecular weight cut-off in the range from 200 to 1000 Da [72,73]. The separation selectivity of NF membranes is mainly attributed to the following three mechanisms: size exclusion, Donnan exclusion (electrostatic repulsion), and dielectric exclusion. For neutral solutes, the retention mainly depends on the size exclusion to reject molecules larger than the size of the membrane pore [74]. The repulsion of the membrane to charged molecules is affected by both size exclusion and Donnan exclusion. The repulsion of the membrane to the ions is affected by Donnan exclusion. When the charged membrane contacts the ionic solution, the ions with the same charge as the membrane are excluded, while the ions with the opposite charge pass through the membrane. It has been proven that the rejection of ions by NF membranes depends on the charge of the membrane and is not affected by the change in the membrane pore size [75,76]. Dielectric exclusion has been the research focus in recent years, but it is still a difficult phenomenon to understand [69,77]. It is generally believed that the mechanism of dielectric exclusion is related to the resistance of the solute penetrating the membrane caused by the energy barrier due to the shedding of the hydrated shell. According to the ion dehydration theory, a lower ion hydration energy can enhance the rejection to achieve effective removal [74,78]. Therefore, contrary to Donnan exclusion, dielectric exclusion is an independent exclusion mechanism [79]. Most NF membranes are thin film composites with a surface ultra-thin porous layer, which is generally prepared by interfacial polymerization and is the key factor to prepare high-performance NF membranes. The ENM possesses high porosity and ultra-thin thickness and is more attractive for the development of NF membranes compared with those fabricated by interfacial polymerization.

**Figure 5:**
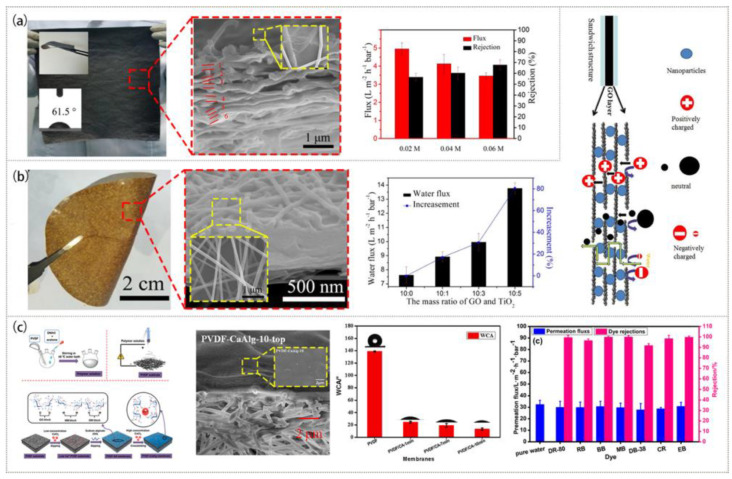
Preparation process, microstructure, and properties of NF membranes: (**a**) GO@Nylon 6 membrane. Reprinted with permission from Ref. [83]. Copyright 2018 Elsevier Ltd. (**b**) (PA 6@GO@PA 6)/TiO_2_ membrane. Reprinted with permission from Ref. [84]. Copyright 2018 Elsevier Ltd. (**c**) PVDF-Alg membrane. Reprinted with permission from Ref. [87]. Copyright 2021 Elsevier Ltd.

Current research focuses on creating stable, effective NF membranes with a high rejection rate and water flux [80,81,82]. Electrospinning and electrospraying were used for the layer-by-layer assembly of a flexible GO mixed Nylon 6 (GO@Nylon 6) multilayer NF membrane (Figure 5a), which realized the locking structure of the multilayer and greatly improved the mechanical stability. It could also maintain a high rejection rate for organic dye and salt ions [83]. On this basis, the PA 6@GO@PA 6 NF membrane mixed with the intercalation of TiO_2_ NPs was prepared through the combination of electrospraying and electrospinning [84]. Figure 5b shows that the pure water flux of the TiO_2_ NPs-PA 6@GO@PA 6 NF membrane was 13.77 L·h^−1^·m^−2^·bar^−1^ at a very low external pressure (1.0 bar) due to the increase in membrane spacing with the insertion of TiO_2_ NPs. Besides TiO_2_, the addition of other NPs, such as SiO_2_, could also make the membrane achieve a high water flux and maintain a high rejection rate [85,86].

In addition, NF membranes have a good decolorization effect on organic dyes in industrial wastewater [87]. As shown in Figure 5c, by impregnating PVDF ENM with NaAlg solution and crosslinking them with CaCl_2_ solution, a unique PVDF-CaAlg NF membrane was created [87]. It had a high rejection rate for several typical dyes, such as 98.5% for methylene blue dye and 99.5% for direct red 80 dye, due to a double membrane composite structure and good surface hydrophilicity.

Due to technical limitations, there is no direct method to prepare NF membranes with several nanopore diameters, and most of them are prepared from ENMs followed by covering polymers on their surface through electrospraying or direct coating. Although these methods can improve the rejection rate of NF membranes for salt ions and other pollutants, this will lead to a great reduction of their water flux. In addition, NF membranes are usually used in high-pressure and complex environments, which is particularly important for the improvement of their mechanical strength and pollution resistance.

#### 2.1.4. Reverse Osmosis (RO)

RO technology has made great progress in the past few decades and accounts for 44% of the world’s seawater desalination productivity [88]. RO membranes, as the core of new seawater desalination technology, are an important research direction. An RO membrane has no distinct pores that directly traverse the membrane. It forms a layered and web-like structure through stacking, so that water must pass through the membrane and reach the permeation side along a tortuous pathway [88]. RO membranes can remove the smallest pollutants (monovalent ions) that are difficult to remove in the NF process. Unlike other pressure-driven membranes, RO membranes have no open channels for pore flow, and transmission is controlled by diffusion. The transport mechanism of RO is called solution diffusion. In this system, water transport is divided into three steps: absorption to the membrane surface, diffusion through the thickness of the membrane, and desorption from the permeate surface of the membrane. RO needs to be driven by applying a pressure greater than the osmotic pressure of the solution during operation, resulting in a positive pressure difference to produce a chemical potential difference (concentration gradient) across the membrane, which makes the liquid cross the membrane against the natural direction of osmosis, while the salts are retained on the influent surface of the membrane [89,90]. Therefore, RO can be used to purify saltwater to produce fresh water on the low-pressure side of the membrane. RO technology has been increasing globally and has gradually replaced the conventional distillation method [89,90,91].

**Figure 6:**
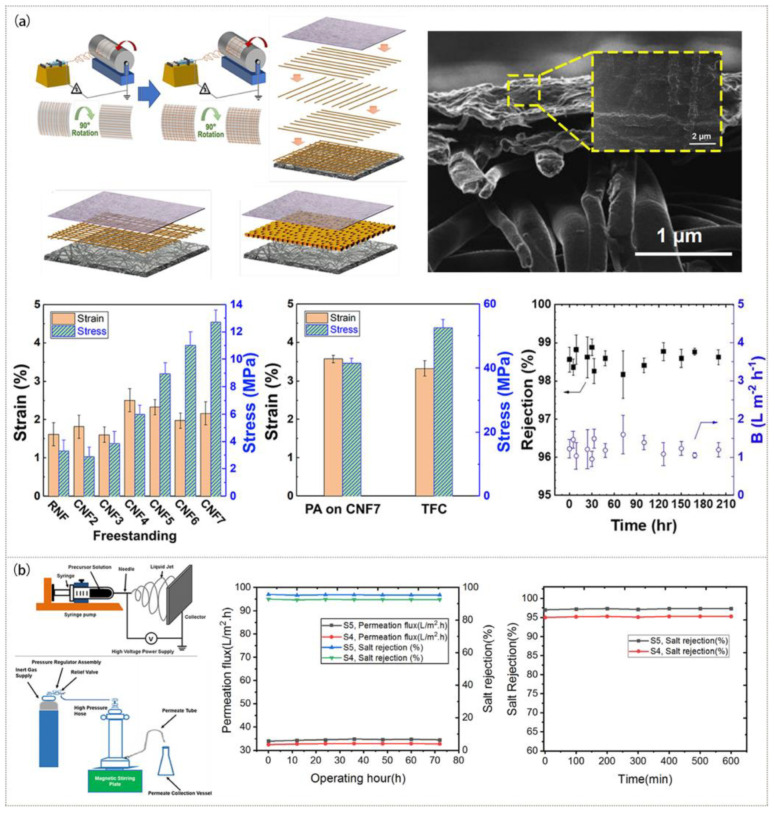
Preparation process, microstructure, and properties of RO membranes: (**a**) PA membrane. Reprinted with permission from Ref. [93]. Copyright 2020 American Chemical Society. (**b**) PVA/ZnO membrane. Reprinted with permission from Ref. [94] Copyright 2019 John Wiley and Sons, Inc.

Generally, RO membranes are composite membranes composed of three layers: an active layer for separation, a microporous support layer, and a non-woven fabric that provides mechanical strength to resist the strong driving pressure required for the RO process [34,92,93]. For example, the non-woven fabric substrate was attached to the electrospun microporous support layer and was then combined with the PA active layer to form a new membrane with a cross structure and nanofiber scaffold by reinforcement and lamination (Figure 6a). When employed in high-pressure seawater desalination operations, the novel structure has good mechanical qualities and can withstand significant pressure [93].

The desalination rate and water flux are also the focus of RO membrane research. In order to obtain a better RO membrane than that made from the conventional method for desalinating saltwater, PVA nanofibers doped with NaAlg and zinc oxide (ZnO) NPs were used as the active layer and deposited on the 3-triethoxysilane-functionalized CA substrate to create a new RO composite membrane, which was finally crosslinked with ethyl orthosilicate (Figure 6b). In this system, the permeation flux could reach 34.6 L·m^−2^·h^−1^ and the desalination rate was 97% [94]. Additionally, the active layer of the RO membrane could also be created by an ENM including CS and maleic acid-crosslinked PVP [91]. By combining the active layer with the 3-triethoxysilane-functionalized CA membrane substrate, another RO membrane was created. The permeation flux and the desalination rate increased to 42.9 L·m^−2^·h^−1^ and 98.3%, respectively, due to the hydrophilicity of the membrane and the negatively charged carboxylate ions generated on the membrane surface.

It is not difficult to find that the influence of the ENM support layer on the surface active separation layer is important in the RO membrane system. In order to guide water molecules through more quickly, the development of an ENM with a large pore size, hydrophilicity, and ultra-thin thickness is the main research direction in preparing high-performance RO nanofiber membranes.

### 2.2. Osmotically Driven Membrane Processes—Forward Osmosis (FO)

FO membrane technology, a separation technique that mimics natural seepage occurrences, is the main kind of osmotic pressure-driven membrane technology. Through the osmotic pressure difference on both sides of the membrane, water molecules selectively pass through, while other substances are rejected on the opposite side. The primary distinction between the FO process and the conventional pressure-driven separation processes is the source of the driving force. Therefore, the FO membrane has greater advantages compared with other membrane separation approaches in some aspects, including a low energy consumption, effective separation performance, a long service life, a wide separation range, and effective separation of high-concentration brine [95,96,97,98].

**Figure 7:**
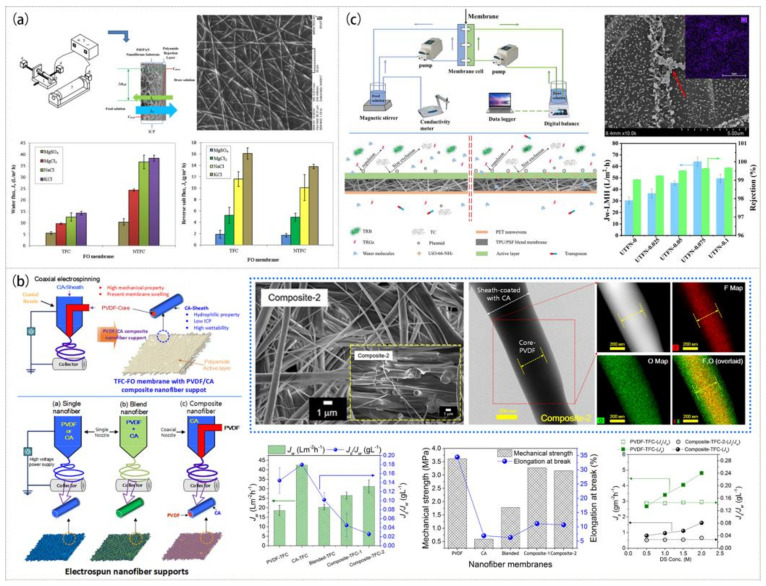
Preparation process, microstructure, and properties of FO membranes: (**a**) PSF/PAN/PA membrane. Reprinted with permission from Ref. [103]. Copyright 2018 Elsevier Ltd. (**b**) CA/PVDF membrane. Reprinted with permission from Ref. [104]. Copyright 2018 Elsevier Ltd. (**c**) TPU/PSF (20/80) membrane. Reprinted with permission from Ref. [106]. Copyright 2020 Elsevier Ltd.

Because of the special driving form, the FO membrane should have a pore structure with high penetration to reduce the impact of internal concentration polarization (ICP) [99,100]. ENM with high porosity is very suitable for the preparation of highly permeable membranes. Recent studies [101,102,103,104] have shown that hydrophilic modification can effectively increase the permeability of the membranes. By coating a layer of PA film over the electrospun PSF/PAN nanofiber substrate through interfacial polymerization, a super hydrophilic PSF/PAN/PA composite membrane was created [103]. The hydrophilicity and water flux of the composite membrane greatly increased and the reverse salt flux (RSF) decreased compared with those of the PSF/PAN composite membrane, as shown in Figure 7a. The composite nanofiber with a PVDF core layer and a CA sheath layer created by coaxial electrospinning was employed as the support layer of the FO membrane to allow good mechanical stability and hydrophilicity, as illustrated in Figure 7b. The water flux of the FO membrane (31.2 L·m^−2^·h^−1^) with the composite nanofiber as the support layer was found to be improved [104].

FO can be effectively used to treat antibiotic wastewater. Some researchers added UiO-66 NPs to a PSF nanofiber membrane by electrospinning and successfully prepared a membrane that could effectively treat antibiotic wastewater [105]. When the content of UiO-66 NPs was 0.5 wt%, the rejection rate for antibiotics reached 99.94% and the water flux reached 50.78 L·m^−2^·h^−1^. UiO-66-NH_2_ NPs obtained by synthesizing 2-aminoterephthalic acid and UiO-66 could also be introduced into nanofibers to create a novel composite membrane with higher water flux. The electrospun TPU/PSF (20/80) membrane was used as the substrate, and hydrophilic UiO-66-NH_2_ NPs were used to modify the surface of the membrane by interfacial polymerization to prepare a nanocomposite FO membrane. It had a superior mechanical strength and could be used to treat antibiotic wastewater with a high rejection rate [106]. Figure 7c shows that the membrane prepared with 0.075 wt% UiO-66-NH_2_ particles had the highest water flux of 64 L·m^−2^·h^−1^, a rejection of more than 99.64% for TC, and a permeability of less than 10% for TRGs.

Although much work on the development of FO nanofiber membranes has been reported in recent years, the low permeation flux and the instability of the surface selective layer still hamper the practical application of FO membranes. Therefore, it is necessary to flexibly use various polymers to prepare ENM as the support layer and combine the surface selection layer with excellent permeability and stability to further optimize the surface and structural morphology of FO membranes. Now, machine learning is emerging in the field of materials, and it is also beginning to cross into the field of electrospinning. The rise of this field may bring important help to the research of FO membrane materials in the future. In addition, how to improve the durability and service life of FO membranes is also an important research direction.

### 2.3. Low Temperature-driven Membrane Process—Membrane Distillation (MD)

MD is a thermally driven process realized by the vapor pressure difference on the surface of the hydrophobic membrane. The basic requirement of MD is that the membrane surface in contact with steam should be hydrophobic to block the solution and make only steam and other gases pass through the membrane. The membrane should also have high porosity to enhance steam penetration to ensure a high water flux. Therefore, the ENM is very suitable for MD due to its high porosity and connected pores [107,108,109,110]. In the past few years, MD has drawn increasing interest from the industries of liquid pharmaceutical concentration, fruit juice manufacture, wastewater treatment, and seawater desalination [111,112,113].

**Figure 8:**
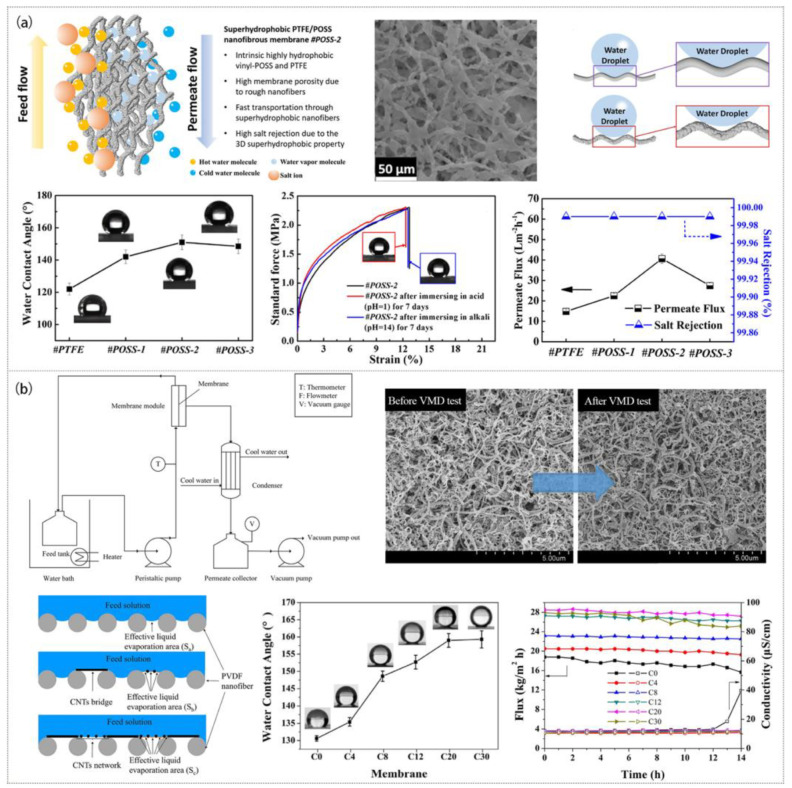
Preparation process, microstructure, and properties of MD membranes: (**a**) PTFE/vinyl- POSS membrane. Reprinted with permission from Ref. [110]. Copyright 2020 Elsevier Ltd. (**b**) PA membrane. Reprinted with permission from Ref. [115]. Copyright 2018 Elsevier Ltd.

MD has four modes, i.e., direct contact membrane distillation (DCMD), vacuum membrane distillation (VMD), sweeping gas membrane distillation (SGMD), and air gap membrane distillation (AGMD) [36]. Among these modes, DCMD is relatively popular and can theoretically provide 100% rejection of non-volatile matter [110,114]. Some researchers incorporated hydrophobic NPs 8-vinyl grafted polyhedral oligosilicone (vinyl-POSS) into electrospun PTFE nanofibers and then calcined them (Figure 8a) to create a composite membrane with superhydrophobicity, high porosity, and good mechanical stability [110]. The addition of vinyl-POSS NPs could increase the crystallization and roughness of PTFE nanofiber membranes, resulting in a high water contact angle of 151.4°. The water flux reached 40 ± 2 L·m^−2^·h^−1^ and salt rejection exceeded 99.99% in DCMD.

Although DCMD has the characteristics of a large flux and high rejection, its direct contact mode will make the heat directly enter the cold side from the hot side, leading to low thermal efficiency and a waste of energy. VMD is a constant-temperature process with little energy loss, so it has received great attention in recent years. During VMD, a large pressure difference will be generated, allowing solutes to enter and block the membrane pores. Therefore, a membrane with a smaller pore size is required to make it have long-term stability [115,116,117,118,119]. Figure 8b illustrates the formation of a thin network layer by the deposition of CNTs on PVDF ENM to construct a super hydrophobic water filtration membrane (CA = 159.3°) with a very small pore size (mean pore size is 0.20 μm) through electrospinning and electrospraying [115]. In the 26 h VMD process, the membrane still maintained a large flux (28.4 L·m^−2^·h^−1^) and a high salt rejection (>99.9%).

MD technology is generally carried out under normal pressure, with low heat demand, energy conservation, and environmental protection. The method is simple in operation and is a low-cost water purification method. In recent years, great progress has been made in the research of new hydrophobic and superhydrophobic ENMs, which have made great contributions to the development of MD membranes. In the future, ENMs with a small pore size and superhydrophobicity should be investigated to make the MD technique more stable and efficient.

## 3. Applications of Nanofiber Membranes in Specific Water Treatment

### 3.1. Membrane Bioreactor (MBR)

In the past few years, MBRs have been designed and applied to treat various pollutants, such as particulate matter, activated pollutants, waste drugs, industrial chemical wastes, and pathogenic microorganisms [120]. These pollutants often cause great harm and can damage the ecosystem and affect biological health. MBR is a mixed system that combines the membrane technique with biological treatment and has higher efficiency, lower space requirements, and a simpler operation process compared with conventional activated sludge treatment plants (CASPs) [121]. MBR does not need the secondary clarifier in CASP and can complete the operation of water treatment through a single-step process. It has been recognized by many countries as a key technology for water recovery and recycling. MBR is usually combined with MF, UF, NF, and RO processes for filtration to meet the sewage discharge requirements after pollutant treatment by using the MBR system [122]. In addition, fresh water suitable for drinking or irrigation can be obtained through the integrated process of combining MBR with the NF/RO system [123]. In recent years, the application of nanofiber membranes in MBR for the treatment of organic contaminants in water has been widely conducted [124,125,126,127].

**Figure 9:**
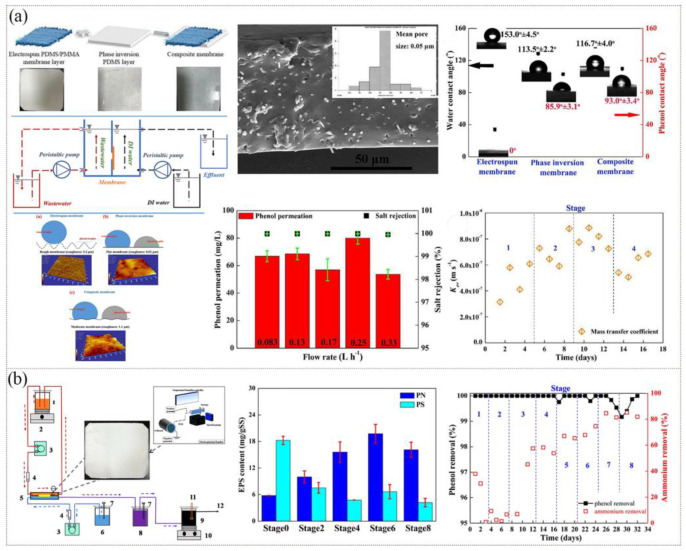
Preparation process, microstructure, and properties of MBR membranes: (**a**) PDMS membrane. Reprinted with permission from Refs. [130,132]. Copyright 2020 Elsevier Ltd., Copyright 2019 Elsevier Ltd. (**b**) PDMS/PMMA membrane. Reprinted with permission from Ref. [131]. Copyright 2020 Elsevier Ltd.

Many studies have found that particulate fouling is easy to deposit onto and into the nanofiber membrane, leading to channel blockage due to the interaction between fouling and the membrane surface, resulting in a significant decrease in MBR performance with the reduction of effective filtration time. Therefore, some measures should be taken to reduce the pollution problem of nanofiber membranes and make them more competitive with the existing commercial film [128,129]. The dry phase inversion method was used to coat PDMS on the surface of electrospun PDMS nanofibers in order to address the issues of low mechanical performance, low mass transfer, and salt leakage of MBR in the treatment of phenol salt-containing waste water (Figure 9a) [130]. Nanofibers were evenly dispersed in the phase inversion layer to create a network of interconnected pores and a loose structure, which made the ENM very easily come into contact with phenol, while its hydrophobic surface could fend off salt incursion. The membrane had a breaking strength of 1.7 MPa and an elongation at break of 60.0%, enabling it to operate stably in MBR. The maximum removal rate of phenol and the conductivity change on the microbial side were 508.9 mg·L^−1^·d^−1^ and 0.1 ms·cm^−1^, respectively, presenting a good degradation effect. In order to achieve the goals of simultaneous phenol infiltration, salt removal, and biodegradation, another study further prepared an electrospun PDMS/PMMA membrane with contact angles of 160.9 ± 2.2° (water) and 0.0° (phenol) [131]. As shown in Figure 9b, phenol solution with a high concentration (290.7 ± 10.4 mg/L) continuously permeated and was completely degraded under a hydraulic retention time of 24 h. Phenol was mostly digested and decomposed by denitrifying bacteria and other heterotrophic bacteria through oxidation.

Compared with the in-depth research progress of nanofiber membranes in other filtration systems, the feasibility of nanofiber membranes in the MBR system is still being studied. The focus of the study is to restore the original working efficiency of the membrane after it is polluted. In addition, it is also necessary to investigate the safety of microorganisms used in MBR. In view of these problems, there will be plenty of space for future research and development.

### 3.2. Heavy Metal Ion Adsorption

Poisoning of water containing toxic heavy metals is one of the most significant global issues due to the rapid development of modern industry and population growth. Chromium, lead, copper, zinc, arsenic, and other heavy metals with carcinogenic, teratogenic, and mutagenic properties are commonly found in water bodies [133,134,135]. Recovering or removing these metal ions from the aquatic environment is extremely important. Currently, various methods including coagulation precipitation, chemical precipitation, adsorption, ion exchange, and membrane separation are used to treat heavy metal ions in wastewater.

The membrane separation method has the advantages of easy use, low cost, and high efficiency. The removal of heavy metal ions can be achieved by adsorption based on ionic interaction between positively charged metal ions and a negatively charged matrix containing functional groups or the formation of coordination bonds between metal ions and the functional matrix through chelation [136]. The ENM has a broad application potential for heavy metal adsorption due to its high porosity, large specific surface area, easy addition of additives, and easy adjustment of surface properties [137,138,139,140]. Table 3 shows some relevant studies on the adsorption of heavy metal ions based on ENM.

**Figure 10:**
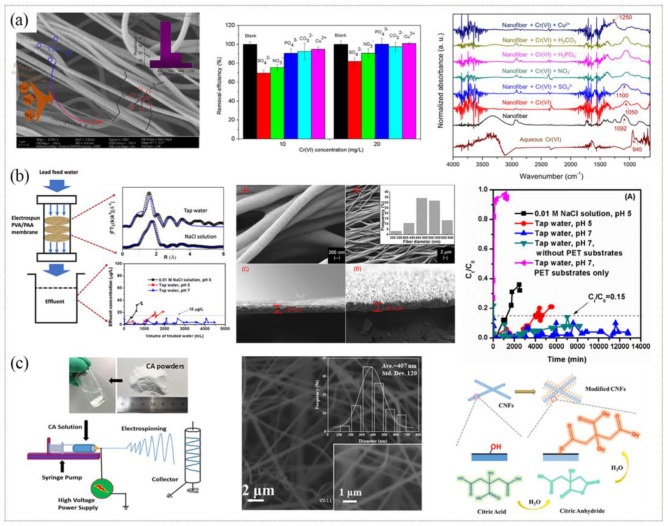
Preparation process, microstructure, and properties of heavy metal ion adsorption membranes: (**a**) PVA/PEI membrane. Reprinted with permission from Ref. [141]. Copyright 2020 Elsevier Ltd. (**b**) PVA/PAA membrane. Reprinted with permission from Ref. [143]. Copyright 2019 Elsevier Ltd. (**c**) Citric acid-incorporated CNFs membrane. Reprinted with permission from Ref. [145]. Copyright 2020 Elsevier Ltd.

The adsorption and removal of heavy metal ions can be realized by mutual attraction between negatively charged functional groups on ENM and metal ions. As shown in Figure 10a, the electrospun PVA/PEI nanofiber membrane could successfully bind hexavalent chromium (Cr(VI)) and separate it from water due to the negatively charged amine groups in PEI, showing good stability and adsorption efficiency. The adsorption of Cr(VI) on nanofiber membranes could reach 150 mg·g^−1^. Moreover, the amine groups in PEI acted as reduction sites after Cr(VI) was adsorbed to reduce Cr(VI) to harmless and chemically stable Cr(III), resulting in the formation of insoluble octahedral Cr(III) oxide [136,141]. Furthermore, the membrane prepared by coating PVA/PVP/PEI ENM with tannic acid had a stronger adsorption capacity for Cr(VI), with the maximum value of 695.04 mg·g^−1^ [142]. The membrane could be calcined into high-purity and harmless CrO_2_ after completing the adsorption of Cr(VI). In addition, a PVA/PAA nanofiber membrane was created to successfully adsorb Pb(II) with high adsorption efficiency and stability (Figure 10b) [143]. The membrane could effectively adsorb heavy metal ions because of the negatively charged carboxyl group in PAA. When the concentration of the Pb(II) solution was 1 mg·L^−1^, the adsorption capacity of the membrane could reach 288 mg·g^−1^, and even after repeated recycling, the nanofiber membrane still had a high adsorption capacity. The eutrophication of water caused by phosphate has attracted widespread attention. A new type of composite membrane was prepared by the in-situ growth of La-doped NiZn-LDH on PAN/GO ENM, which can effectively adsorb phosphorus-containing wastewater [150]. Interestingly, some researchers found that the use of phosphate-functionalized PAN ENM could effectively remove U(VI) in water due to the adsorption of hydroxyl groups in the phosphate for U(VI) [144]. This discovery helped to promote the technology of the rapid detection and removal of uranium in water.

It is also an important method to adsorb heavy metal ions by chelating them to the functional matrix on ENM [136,145,151]. Citric acid chelating agents are widely used in the adsorption of heavy metal ions. Some researchers prepared functionalized cellulose nanofibers through deacetylation and citric acid modification [145], which showed a high adsorption capacity for Cr(VI) (Figure 10c). The cumulative toxicity of As(V) in the environment is a matter of concern. Some researchers have fixed hydrophilic L-cysteine biomolecules into PVA ENM to form a complex for the effective adsorption of As(V) in water [152]. Furthermore, the nanofiber membrane prepared with MOF-808 and GO as the main raw materials has many exposed adsorption sites, which can effectively adsorb As(V) and Hg(II) in water [146,147]. In addition, sepiolite can provide a wide range of potential applications in drinking water treatment, adsorption, and other fields. A new type of nanofiber membrane could be prepared by combining this material with PU through electrospinning to effectively remove heavy metal ions in water [148]. This nanofiber membrane had a high chelating adsorption capacity for Cr, Cd, As, and Mn due to the existence of the polar oxhydryl groups on sepiolite.

The development of multifunctional ENM provides a new technical means for the adsorption of heavy metal ions. In recent years, researchers have developed various ENMs to effectively adsorb different heavy metal ions and have made encouraging progress. However, it must be pointed out that due to the strong adsorption capacity of ENM for heavy metal ions, its desorption capacity is slightly insufficient, which will affect the recycling capacity of ENM. Therefore, it is necessary to further study the relationship between the adsorption capacity of ENM and various environmental conditions in order to better understand the adsorption–desorption process of ENM to improve the adsorption stability, recyclability, and recoverability of ENM.

### 3.3. Oil–water Separation

Due to the increasing output of oily effluent in the food and petrochemical industries and the frequent occurrence of oil leakage, oil pollution has become one of the issues of global concern. Oily substances are typically poisonous and water insoluble. Conventional oil–water separation technologies including coagulation, air flotation, and chemical degradation generally have the disadvantages of a complex operation and waste of energy [153,154]. However, membrane separation technology is a straightforward and energy-saving process exhibiting excellent separation performance. Nanofiber membranes are considered particularly good oil–water separation materials, which can be divided into hydrophobic/oleophilic membranes and oleophobic/hydrophilic membranes [155,156,157]. Table 4 shows the characteristics of some oil–water separation membranes.

The hydrophilic/oleophobic membrane can effectively separate oil from water by allowing water to pass through while blocking oil during filtration. The electrospun PES/PVP nanofiber membrane with Fe_3_O_4_ NPs and n-methylpyrrolidone was extremely hydrophilic with a water contact angle of only 21.78 ± 0.3°, which endowed it with an outstanding oil removal performance of 94.01% and an excellent water flux recovery rate of 79.50% [158]. Moreover, the nanofiber membrane had good oil resistance and flux stability due to its surface resistance-mediated effect. Furthermore, the addition of ZIF-8 into the electrospun nylon 6,6 nanofiber membrane was reported to increase its mechanical strength and permeability [159]. This nylon 6,6/ZIF-8 membrane could achieve a high oil rejection of 89%, which was several times higher than that for pure nylon 6.6 membrane. The oil–water separation membrane prepared by adding ZnO NPs to nylon 6 nanofibers by electrospinning had an oil removal rate higher than 90% [166]. Moreover, the membrane had an antifouling performance with a flux recovery rate higher than 52.5%. Some researchers also prepared a new controllable oil–water separation ENM by combining CA and poly(N-isopropyl acrylamide) to form a thermal-responsive polymer membrane [160]. The membrane shows strong hydrophilicity and can be used for rapid oil–water separation at room temperature. When the temperature rises, the membrane shows a strong hydrophobicity, and the separation process stops.

The hydrophobic/oleophilic membrane can effectively separate oil from water by allowing oil to pass through and blocking water during filtration. The Cu_2_O NPs were deposited on a melt-electrospun PP fibrous membrane through magnetron sputtering to create a PP/Cu_2_O composite membrane with superhydrophobic (water contact angle of 151.8 ± 2.1°) and super oleophilic (oil contact angle of 0°) characteristics [161]. The separation efficiency of the composite membrane for oil–water emulsions exceeded 92%, and that for the n-hexane/water mixture was 94.14%. Furthermore, the separation efficiency remained at 92.52% after 30 cycles of filtering with good stability due to the strong adhesion between Cu_2_O NPs and the fiber membrane. Additionally, PVDF/rGO/TiO_2_ nanofibers were prepared through electrospinning by incorporating rGO/TiO_2_ NPs into PVDF solution [162]. The membrane exhibited certain hydrophobicity (water contact angle of 100°) and excellent lipophilicity (oil contact angle of 0°) with the addition of rGO/TiO_2_ and had an oil removal efficiency of 98.46%.

However, in the face of large-scale oil leakage, direct filtration is often difficult to deal with such oil pollution, and the method of adsorption–desorption using a hydrophobic/oleophilic membrane is often convenient and fast [163,167]. The PET ENM can effectively adsorb crude oil, diesel oil, gasoline, and pump oil, etc., and can be squeezed to recover oil for recycling [163]. The research shows that the adsorption capacity is still higher than 75% after five times of repeated use. In addition, biodegradable PLA ENM is a promising environmental protection membrane material, which shows an excellent adsorption performance for lubricating oil, diesel oil, and edible oil and still maintains this performance after ten recycling events [165]. Hydrophobic PI ENM has strong adsorption properties for crude oil (61 g·g^−1^) and diesel oil (109 g·g^−1^) and can be used to recover oil by mechanical extrusion and maintain the initial adsorption capacity of more than 80% after repeated use [164]. This strong adsorption and recycling performance shows great application potential when oil leakage pollution occurs. In addition to being used for the adsorption of oil, the PI ENM also has a strong adsorption on dyes [168]. Therefore, it has broad development prospects in environmental applications.

As described in this section, ENM shows significant advantages and excellent performance in oil–water separation. However, problems such as insufficient mechanical properties, serious membrane pollution, and a complex manufacturing process still exist. In particular, plugging of the membrane pores will occur and cause considerable trouble if the membrane surface is adsorbed by oil droplets and is difficult to desorb during use. In serious cases, it may even cause irreversible damage to the membrane. Therefore, it is necessary to pay more attention to adsorption–desorption, the anti-adhesion performance, and the surface stability of the ENM for oil–water separation in the future.

### 3.4. Photocatalysis

The highly concentrated chemical waste water generated in an industrial manufacturing process will pollute the natural water source and exert a significant negative impact on the ecological environment and human health. During membrane treatment, many substances cannot be completely filtered or absorbed by the fiber membrane. Therefore, one of the key research areas of nanofiber membranes is to degrade pollutants by integrating photocatalytic processes on the filter membranes [169,170,171,172].

Previously, the photocatalysis method was usually to directly mix the powdered photocatalysis materials with sewage for decomposition, while it was difficult to recover the photocatalysis materials, resulting in a waste of resources and secondary pollution [169,171]. In order to promote catalysis, photocatalytic materials can be fixed on electrospun nanofibers, which can make catalysis work well because of their large specific surface area, and the photocatalytic materials can also be easily recycled [169,173,174]. Some metal oxides, including CuO, ZnO, and TiO_2_, have narrow band gaps, easily produce a light response, and have outstanding photocatalytic properties. Therefore, they are frequently coupled with nanofibers to break down organic contaminants through photocatalysis [173,174,175,176]. TiO_2_ has become the most widely used photocatalyst material due to its chemical stability, corrosion resistance, non-toxicity, and low cost. Moreover, TiO_2_ nanofibers were also found to be more effective than TiO_2_ NPs in removing pollutants. By carbonating electrospun PAN/Tetrabutyl titanate nanofibers, a TiO_2_/N-doped carbon nanofiber (TiO_2_/NCNF) membrane with memory catalytic characteristics was created [174]. Memory catalysis is where graphitized carbon can accept electrons generated by TiO_2_ under light irradiation and store some of them and then release electrons in a dark environment to keep the reaction going. TiO_2_ is uniformly distributed and firmly fixed on the ultra-long nanofibers, enhancing the mechanical and flexible properties of the membrane. This self-supporting TiO_2_/NCNF membrane could provide more active sites and completely eliminate contaminants during processing without centrifugation and filtration. Additionally, a Ag/TiO_2_-doped PVP nanofiber membrane with good photocatalytic degradation activity for phenol under visible light was created by electrospinning [169]. The results showed that the phenol concentration, pH value, and catalyst dosage had significant effects on the degradation of phenol, and the degradation rate could reach up to 92.91%.

The efficacy of using an ENM in conjunction with photocatalytic technology to break down organic contaminants in wastewater has been fully demonstrated by the current research. A nanofiber membrane immobilizes the photocatalyst materials, which can be easily and quickly recycled to eliminate secondary pollution. At present, the limitation of photocatalysis membranes lies in the fact that pollutants remain on the membrane surface during the degradation process, hindering the light source and affecting the reaction. Therefore, more research and discussion are needed to improve the stability, applicability, and durability of the membranes.

## 4. Antifouling Technology of Nanofiber Membranes in the Water Treatment Process

### 4.1. Antifouling Modification of Nanofiber Membranes

A contaminated filter membrane for water treatment can diminish the filtration efficiency and deteriorate the water quality [177,178,179]. In recent years, extensive research has been carried out to optimize the antifouling performance of the membrane by altering the hydrophilicity, the Zeta potential, and the roughness of the membrane surface [180,181,182]. Table 5 summarizes some common optimization methods for antifouling and their effects.

**Figure 11:**
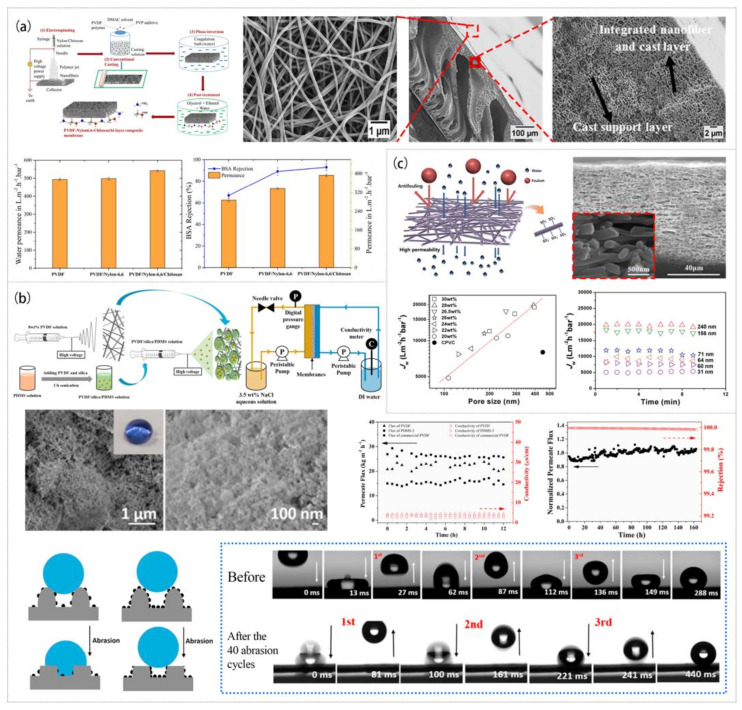
Preparation process, microstructure, and properties of related antifouling membranes: (**a**) PVDF/nylon-6,6/CS nanofiber UF membrane. Reprinted with permission from Ref. [183]. Copyright 2017 American Chemical Society. (**b**) PVDF/PDMS/silica MD membrane. Reprinted with permission from Ref. [186]. Copyright 2020 Elsevier Ltd. (**c**) PVDF/Nafion nanofiber NF membrane. Reprinted with permission from Ref. [187]. Copyright 2014 American Chemical Society.

In most cases, the hydrophilicity of the membrane will significantly affect the antifouling ability of the membrane. Figure 11a shows the two-step process used to create a Janus nanofiber composite UF membrane, which involved electrospinning a hydrophilic nylon-6,6/CS nanofiber membrane and coating the surface with PVDF solution [183]. The irreversible fouling rate of the PVDF/nylon-6,6/CS membrane was lowered by 78% and the reversible fouling rate was 2.2 times higher than that of the pure PVDF membrane after 4 h of filtration with BSA foulants due to the reduction of the water contact angle by 72%. In addition, a self-supporting hydrophilic nanofiber membrane could be obtained by mixing f-PPTA with PVDF followed by electrospinning [184]. The f-PPTA-containing hydrophilic chains migrated to the nanofiber surface due to its self-migration and electric field induction, which greatly improved the wettability of the whole membrane and accelerated the process of water molecules passing through the membrane. The flux and BSA rejection were maintained at approximately 89% and 99%, respectively, of the initial values after 6 h of operation. A composite membrane was formed by compounding PVDF with thermo-responsive attapulgite (t-ATP), and its surface was then modified by poly(N-isopropylacrylamide) (PNIPAM) to make a membrane with strong wettability [188]. The surface PANIPAM expands or contracts with the change of temperature to flexibly release the biological dirt in the membrane, showing excellent anti-biological pollution performance. It was found that 89% of the surface dirt could be removed through simple temperature change and ultrasonic cleaning. The nanofiber membrane prepared by PES combined with amphiphilic polyethyleneoxide/polypropyleneoxide multiblock copolymers had a good hydrophilicity [182]. Its water flux loss was reduced from 68% to only 34%, showing an excellent antifouling performance. Therefore, a membrane with more hydrophilic chains can improve the antifouling ability of the membrane and prevent the fouling of the membrane surface and internal pore structure.

However, membrane pores are transport channels of substances other than water for some types of membrane (e.g., MD and oil–water separation membranes); consequently, pore wetting seriously affects the membrane filtration process, leading to the fouling of the membrane. Therefore, it is required that the membrane should have excellent hydrophobicity to improve its antifouling ability. The dense membrane made by combining electrospun PVDF nanofibers and electrosprayed PVDF/PDMS/silica powders exhibited excellent superhydrophobicity with a water contact angle of 170.1° as shown in Figure 11b [186]. It also had an unusual wear resistance and could remain superhydrophobic after 40 wear cycles. In addition, a new high-performance MD membrane was prepared by using electrohydrodynamic techniques. The membrane combines the electrosprayed super-hydrophobic surface layer, the intermediate layer of electrospun hydrophobic nanofibers, and the substrate layer of the hydrophilic microporous membrane [189]. The surface layer prepared by electrospraying the PVDF solution containing SiO_2_ has strong hydrophobicity, excellent antifouling, and a strong resistance to wetting. The middle layer of electrospun hydrophobic PVDF nanofibers is conducive to the rapid passage of water because of the high porosity. In DCMD, no obvious fouling and wetting were observed even at 60% water recovery. This study provides an effective method for the preparation of high-performance MD membranes and has practical application potential in the field of high concentration brine treatment. In terms of oil–water separation, the strong hydrophilicity of the membrane will lead to the wetting of membrane pores and will affect the effect of oil–water separation [190,191,192]. Some researchers prepared a hydrophobic PVDF/SiO_2_ nanofiber membrane inspired by the surface structure of lotus leaves to reduce membrane pollution [190]. This biomimetic membrane can effectively treat water-in-oil emulsion and completely recover the initial flux after cleaning. In addition, the environmentally friendly ENM prepared by mixing bamboo and PAN solution stably separated various oil-in-water emulsions [191]. Furthermore, the new 6FDA-TrMPD (formed by the condensation of 4,4’-(hexafluoroisopropylidene) diphthalic anhydride (6FDA) and 2,4,6-trimethyl-m-phenylenediamine (TrMPD) at high temperature) ENM and the porous PAN/PVP ENM treated by water erosion both showed excellent oil–water separation characteristics [192,193]. These membrane materials provide a new way for the development of sewage purification in the future.

In addition, charge exclusion is also an effective means to prevent membrane fouling [187]. The negatively charged PVDF/Nafion nanofiber membrane prepared by electrospinning showed excellent water permeability and antifouling properties as shown in Figure 11c [187]. The presence of sulfonate groups in the fiber enhances the antifouling performance of the membrane against oil and the pollutants with negative electricity. The high porosity (80%) of the nanofiber membrane makes it have good water permeability, which also contributes to the antifouling performance. In addition, a negatively charged hydrophilic nanofiber membrane was successfully prepared by mixing sulfonated PVDF (S-PVDF), PVDF, and GO followed by electrospinning [194]. The S-PVDF/PVDF/GO membrane had a high anti-pollution ability against oily and negatively charged pollutants due to the electrostatic repulsion and exhibited a 70% decrease in the degree of pollution compared with a pure PVDF membrane under the same experimental conditions.

### 4.2. Self-Cleaning and Flux Recovery of Nanofiber Membranes

In order to maintain stable water filtration after membrane pollution, conventional water filtration membranes typically use rinsing or direct replacement, which might lead to further pollution and material waste [195,196]. Some new nanofiber membranes can achieve self-cleaning to restore the membrane flux by the addition of outside energy. According to the different forms of external energy input, they are mainly divided into two types of membranes: one is a photocatalytic membrane, which uses visible light or ultraviolet light to generate reactive oxygen species to degrade organic substances inside and outside the membrane; the other is an electrocatalytic membrane, which serves as an electrode and performs electrocatalytic removal of pollutants [197,198,199].

**Figure 12:**
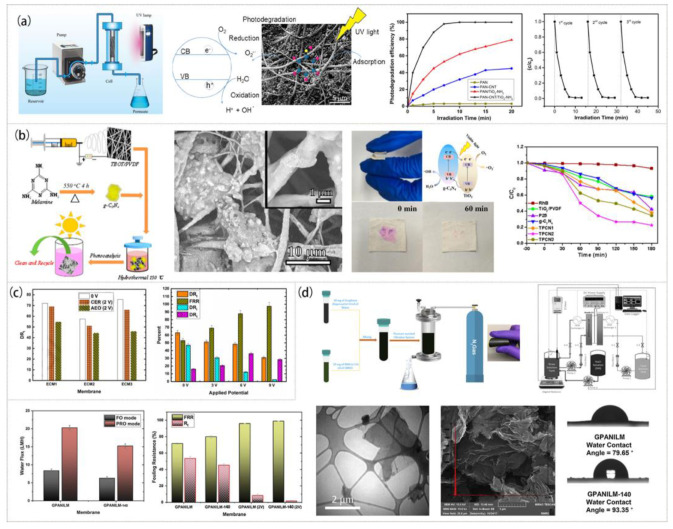
Preparation process, microstructure, and properties of membranes with the flux recovery self-cleaning function: (**a**) PAN-CNT/TiO_2_-NH_2_ membrane. Reprinted with permission from Ref. [200]. Copyright 2020 Springer Nature. (**b**) TiO_2_/PVDF/g-C_3_N_4_ membrane. Reprinted with permission from Ref. [201]. Copyright 2020 Multidisciplinary Digital Publishing Institute. (**c**) PES-PANI/rGO conductive membrane. Reprinted with permission from Ref. [196]. Copyright 2020 Elsevier Ltd. (**d**) PAI-PANI/rGO conductive membrane. Reprinted with permission from Ref. [202]. Copyright 2019 Elsevier Ltd.

In the case of self-cleaning photocatalytic film, TiO_2_ as a widely used high-performance photocatalytic nanomaterial could be combined with a variety of film materials with good light transmittance [169,174,200]. Figure 12a displays the stable photodegradation capability of the PAN-CNT/TiO_2_-NH_2_ composite nanofiber membrane to phenol under UV light irradiation [200]. The photodegradation rate of the nanofiber composite membrane could reach 99% within 7 min, and its catalytic performance did not decrease significantly after three cycles, showing good reusability. Moreover, the membrane flux could also be recovered well. In addition, the synthesized TiO_2_/g-C_3_N_4_ particles were tightly embedded in the PVDF nanofibers to prepare a self-supporting membrane by electrospinning. The membrane exhibited remarkable photocatalytic activity and self-cleaning ability under visible light and could overcome secondary pollution (Figure 12b). Compared with the traditional TiO_2_ powder photocatalyst, a combination of TiO_2_ with the nanofiber membrane could make it easier to recycle and has a good development prospect in the field of photocatalysis [201].

Membranes have the advantages of a high specific surface area and rich active sites, which make them more conductive than bulk materials. Their unique fiber structure provides a path for the rapid transport of carriers, so they can effectively conduct electrocatalysis [196,197,202]. The PANI membrane containing rGO and the PES membrane were joined using pressure-assisted technology to produce an electrocatalytic membrane (PES-PANI/rGO) [196], which was subsequently used as the anode in studies on NaAlg fouling. The cleaning experiment showed that the membrane fouling rate was reduced by more than 30% and the flux recovery rate was positively correlated with the applied voltage. Some other researchers created a PAI-PANI/rGO electrocatalytic membrane using basically the same preparation method [202]. As shown in Figure 12c,d, effects of PAI and PES on the performance of the above two kinds of electrocatalytic membrane were compared. It was found that the flux recovery rate of the PAI-PANI/rGO membrane reached 79.9% and then increased to 98.8% when the applied voltage was 2 V after the membrane was cleaned with ultrapure water. For PES-PANI/rGO, the flux recovery rate was only 54.5%, which then reached 97.5% when the applied voltage was 9 V. In contrast, the recovery rate of an ordinary PES membrane after cleaning with ultrapure water was only 21.8%.

## 5. Conclusions and Future Perspectives

### 5.1. Conclusions

For a long time, water safety has been a key issue attracting worldwide attention. Various water treatment technologies have been developed to solve the problem of water pollution. The application of nanofiber membranes in water treatment provides a new dimension for this field. They can greatly improve the efficiency of water treatment technology because of their good material compatibilities, high specific surface area, high porosity, flexibility, and manufacturing simplicity. Electrospinning, as a simple method to prepare nanofibers, has received widespread attention. In this review, we briefly summarized the principles and existing problems of various membrane technologies and introduced the research trends of various nanofibers used for water purification. Finally, the anti-fouling methods of membranes and the means of membrane flux recovery were also discussed.

Although many research achievements have been made in ENM used for water treatment, it is still necessary to further develop nanofiber membranes with ultra-fine fiber size, high porosity, and excellent selectivity, create solid and ultra-thin active layers on nanofiber substrates, and construct complex membrane structures. In addition, making the water filtration membrane more functional through surface coating is also the main research direction. The research in this field mainly focuses on the reinforcement of matrix materials, the selection of coating materials, and the methods of bonding between the matrix and coating. The addition of various NPs can also endow the membrane materials functions such as photocatalysis and can expand the application field of water filtration membranes. This may have a certain impact on the porosity, roughness, electrical performance, and selective permeability of the membranes. In fact, the main challenge of membrane treatment technology is the reduction of filtration efficiency caused by membrane structure damage or pollution in complex use environments. The mechanical strength of the membrane can be effectively improved by means of modification and combination to improve the stability of the membrane. In terms of pollution resistance, it is an effective solution to directly resist the invasion of pollutants or self clean pollutants to restore membrane flux. In the future, various modification methods should be attempted to prevent the accumulation of contaminants on the surface and inside of the membrane.

### 5.2. Future Perspectives

With the shortage of water resources and the increase in water-borne diseases, human beings are facing great pressure to improve water quality. Researchers have used various types of ENMs or their composites as the most effective membrane materials in filtration applications. The surface functionalization of nanofiber membranes with a porous structure determines their permeability, application direction, cost, efficiency, and service life. The main research directions of these nanofiber membranes in the future will focus on long-term stability, low cost, catalytic degradation, antifouling performance, and mass production. In addition, the use of ultrafine nanofibers less than 100 nm can effectively reduce the pore size of the membrane and obtain high porosity, which can improve the invasion of small particles without significantly sacrificing water flux. Therefore, the stable preparation of finer nanofibers will bring an exciting influence to membrane water treatment technologies.

In recent years, environmental protection, energy conservation, and renewability have attracted more and more attention in various fields. In the future, electrospinning technology will be more widely applied in the development of economic and ecological nanofiber membranes for water treatment, and its application in low-energy consumption water treatment methods such as FO, MD, and MBR will also be the focus of attention. However, as a water treatment technology with low energy consumption, low pollution, and wide application, FO cannot be widely used because of its own problems. Therefore, we should focus on the selection of materials and the structure design of composite membranes to obtain efficient FO membranes. In recent years, the application of machine learning in material science has increased, and electrospinning technology is also involved in this field, which can help the water treatment field to find the best new membrane materials conveniently and rapidly. In general, the development of ENMs with multifunctionality and a reasonable structure will become the main solution for effective water treatment. It is expected that water treatment technology based on ENMs will have greater development and better application in the future.

## Figures and Tables

**Table 1:** Abbreviations.

Abbreviation	Full Name	Abbreviation	Full Name
MF	Microfiltration	PA 6	Polyamide 6
UF	Ultrafiltration	CS	Chitosan
NF	Nanofiltration	ZIF-8	Zeolitic imidazolate framework-8
RO	Reverse osmosis	CNTs	Carbon nanotubes
FO	Forward osmosis	PA	Polyamide
MBR	Membrane bioreactor	CA	Cellulose acetate
MD	Membrane distillation	TPU	Thermoplastic polyurethane
ENM	Electrospun nanofiber membrane	PSF	Polysulfone
PASS	Poly(arylene sulfide sulfone)	TC	Tetracycline
PVC	Polyvinyl chloride	TRGs	Tetracycline resistance genes
PVDF	Polyvinylidene fluoride	PDMS	Polydimethylsiloxane
PVA	Polyvinyl alcohol	PMMA	Polymethyl methacrylate
PEEK	Polyetheretherketone	PEI	Polyethylenimine
PE	Polyethylene	PVP	Polyvinylpyrrolidone
PP	Polypropylene	PES	Polyethersulfone
PHB	Polyhydroxybutyrate	rGO	Reduced graphene oxide
PTFE	Poly tetra fluoroethylene	PANI	Polyaniline
CNFs	Cellulose nanofibers	PAI	Polyamide-imide
PAA	Poly(acrylic acid)	PET	Polyethylene terephthalate
PI	Polyimide	PLA	Polylactic acid
PEO	Polyethylene oxide	NaAlg	Sodium alginate
PAN	Polyacrylonitrile	CaAlg	Calcium alginate
MOF-808	Zr-based metal organic framework	f-PPTA	Fluxible poly(p-phenylene) terephthalamide)

**Table 2:** The characteristics of different membrane processes based on ENM.

Membrane Processes	Common Materials for Electrospinning	Size of Contaminants	Advantages	Existing Challenges and Limitations	Refs.
MF	PAN, PVDF, PA, PE, PEEK, PP, etc.	0.1–10 μm	High porosity; high flux; pore connectivity	Large aperture; easy to be blocked and polluted	[8,31]
UF	PAN, PVDF, PES, PEEK, PSF, etc.	0.01–0.1 µm	High permeate flux; asymmetric microporous structure	Sensitive to pressure; difficulty in cleaning	[32]
NF	PVDF, PSF, CA, PA, PHB, etc.	0.001–0.01 µm	Strong performance for desalination; high retention of divalent ions	High cost; instability in the selective layer	[33]
RO	PA, PVA, CA, PVP, CS, etc.	0.0001–0.001 µm	High efficiency; strong separation ability	High pressure requirements; membrane fouling; susceptible to temperature	[34]
FO	PAN, PSF, PES. etc.	0.0001–0.001 µm	Low membrane fouling; high rejection; energy conservation	Low flux; difficult to recycle the draw solution	[35]
MD	PVDF, PTFE, PAN, etc.	0.0001–0.001 µm	Low energy consumption; low-cost seawater desalination	Serious heat loss; membrane wetting	[36]

**Table 3:** Several studies on heavy metal ion adsorption based on ENM.

Membrane Materials	Adsorption Principles	Types of Adsorbed Heavy Metal Ions	Refs.
PVA/PEI ENM	Adsorbed through amino functional groups	Cr(VI)	[141]
PVA/PVP/PEI ENM coated with tannic acid	Adsorbed through amino functional groups	Cr(VI)	[142]
PVA/PAA ENM	Adsorbed through carboxyl functional groups	Pb(II)	[143]
Phosphate-functionalized PAN ENM	Adsorbed through hydroxyl groups	U(VI)	[144]
Cellulose ENM obtained through deacetylation and citric acid modification	Adsorbed through a citric acid chelating agent	Cr(VI)	[145]
ENM prepared with MOF-808	Adsorbed through the adsorption sites brought by MOF-808	Hg(II); As(V)	[146,147]
PU/sepiolite ENM	Adsorbed through polar oxhydryl groups	Cr(VI), Cd(II), etc.	[148]
Wool keratin/PA 6 ENM	Adsorbed through free carboxyl groups in keratin	Cu(II)	[149]

**Table 4:** Several studies on oil–water separation based on ENM.

Types of Oil–Water Separation	Membrane Materials	Types of Emulsion or Oil	Refs.
Direct filtration; hydrophilic/oleophobic membrane	PES/PVP ENM added with Fe_3_O_4_ NPs and n-methylpyrrolidone	Oil/water emulsion; synthetic oil	[158]
	Nylon 6,6/ZIF-8 ENM	Oil/water emulsion	[159]
	CA/poly(N-isopropyl acrylamide) ENM	Oil/water emulsion	[160]
Direct filtration; hydrophobic/oleophilic membrane	Melt-electrospun PP fibrous membrane deposited by Cu_2_O NPs through magnetron sputtering	Oil/water emulsion; bean oil; motor oil; petroleum oil;n-hexane	[161]
	PVDF/rGO/TiO_2_ ENM	Oil/water emulsion	[162]
Oil adsorption; hydrophobic/oleophilic membrane	PET ENM	Crude oil; diesel oil; gasoline; pump oil, etc.	[163]
	PI ENM	Crude oil; diesel oil; gasoline	[164]
	PLA ENM	Lubriacnt oil; diesel oil; cooking oil	[165]

**Table 5:** Research on antifouling performance based on ENM.

Materials	Additives	Applications	Optimization Methods	Antifouling Performance	Refs.
PVDF	Nylon-6,6/CS	UF	Superhydrophilicity	Compared with PVDF membrane, the irreversible fouling rate decreased by 78%.	[183]
PVDF	f-PPTA	UF	Superhydrophilicity	It maintained approximately 89% of the initial flux after 6 h of operation.	[184]
PAN	GO NPs	MF	Hydrophilicity	A high flux recovery ratio of 96.6% and a low irreversible fouling ratio of 3.4% were obtained.	[185]
PVDF	PDMS/silica fume	MD	Superhydrophobicity	It maintained a stable water flux and achieved a rejection rate higher than 99.99% during 160 h of operation.	[186]
PVDF	Nafion	Oil proof	Static electricity	The recovered water permeability percentages for the membranes were 30% and 24%.	[187]

## Data Availability

Data presented in this study are available on request from the corresponding author.
